# Knowledge about the Risk of Cardiovascular Disease among Adults with Type 2 Diabetes Visiting the Outpatient Clinics at Tertiary Hospital in Riyadh, Saudi Arabia: A Cross-Sectional Study

**DOI:** 10.3390/ijerph19094996

**Published:** 2022-04-20

**Authors:** Shatha A. Alduraywish, Fahad M. Aldakheel, Lojain A. Azizalrahman, Lujain T. Alzaid, Shouq S. Alqahtani, Sara H. Alhussein, Atheer M. Almutairi

**Affiliations:** 1 Department of Family and Community Medicine, College of Medicine, King Saud University, Riyadh 11461, Saudi Arabia; 2 Department of Clinical Laboratory Sciences, College of Applied Medical Sciences, King Saud University, Riyadh 11433, Saudi Arabia; faldakheel@ksu.edu.sa; 3 College of Medicine, King Saud University, Riyadh 11461, Saudi Arabia; lazizalrahman@gmail.com (L.A.A.); lalzaid3@gmail.com (L.T.A.); shouq.35@gmail.com (S.S.A.); sara.hus.g8@gmail.com (S.H.A.); atheermbs17@gmail.com (A.M.A.)

**Keywords:** cardiovascular risk, Heart Disease Fact Questionnaire, health knowledge, prevention, type 2 diabetes mellitus

## Abstract

Background: One of the primary goals of diabetes management is to prevent cardiovascular events. The rate of cardiovascular diseases (CVDs) is significantly high in the diabetic population. Inadequate knowledge of CVDs risk in diabetes may result in the failure of its early prevention, causing increased morbidity and mortality. The objectives of this study were to assess the CVD risk knowledge and determine the predictors of the knowledge among adults with type 2 diabetes. Methods: A cross-sectional study was conducted to examine the study objectives. Convenience sampling was used to recruit adults with type 2 diabetes mellitus (DM) who visited the King Saud University Medical City (KSUMC) outpatient clinics. Data were collected using the Heart Disease Fact Questionnaire. SPSS software (version 24.0) was used for data analysis. Results: A total of 383 patients were recruited. The level of CVD risk knowledge was high among all participants (19.04 ± 3.47). There were significant differences in knowledge scores between different groups, including age (*p* = 0.01), marital status (*p* = 0.01), and type of residence (*p* = 0.04). Participants who were older than 40 years, married, and lived in traditional houses had higher knowledge scores. Conclusion: The study findings indicated a high level of CVD risk knowledge in our study population. The presence of multidisciplinary intensive education programmes targeting type 2 DM patients, such as that conducted at KSUMC, might be responsible for the higher levels of knowledge among our study population. Thus, increasing the efforts towards the education of patients will improve the level of knowledge, including CVD risk knowledge.

## 1. Introduction

Cardiovascular diseases (CVDs) are the main cause of morbidity and mortality in individuals with diabetes [1]. The risk of developing CVDs is two-to-four times higher in individuals diagnosed with diabetes than in others [2]. Inadequate knowledge of CVD risk in diabetes may lead to the failure of its early detection and prevention. Thus, it is important that individuals with diabetes have sufficient ‘risk knowledge’ of CVDs [3].

The 2018 Household Health Survey conducted in Saudi Arabia reported that approximately 10% of the Saudi population aged >15 years were diagnosed with diabetes, among which 17% and 13% were estimated to have coronary artery disease and stroke, respectively [4]. Despite the high incidence of CVDs among diabetic populations and the clear correlation between diabetes and increased CVDs risk, not all patients with diabetes are aware of the cardiovascular risk and other diabetes-related complications [5].

Several studies from 2014 to 2019 examined the knowledge of CVD risk among patients with diabetes. Most studies were conducted in developing countries [6,7,8,9,10,11,12,13]. These studies had varied results, with some studies showing that the knowledge of cardiovascular complications in patients with diabetes was lower than 50%. A 2014 study in India6 showed that only 44% of the respondents reported heart disease as a possible complication of diabetes. Furthermore, a study in Ghana [7] revealed that 80% of the participants lacked knowledge of heart disease risk in diabetes.

Conversely, other studies found that the level of knowledge of CVD risk was high (≥50%) among the study population. Another study conducted in India [8] indicated that 89% of patients with diabetes were knowledgeable about macrovascular complications, which was contradictory to the findings of the previously mentioned study that was also conducted in India [6]. Major differences, mainly related to the sample size, study population, and study design, may have contributed to the two opposite outcomes.

A study in Pakistan [9] also revealed lower levels of CVD risk knowledge (50–60%). In Nigeria [10], a study showed that, although awareness of diabetic complications was high (90.5%), the specific knowledge of cardiac complications was only 61.9%. These results were consistent with a Turkish study in which approximately 62.81% of the study population had knowledge of CVD risk [3]. A more recent study conducted in Ethiopia in 201,911 reported that 63.2% of the participants had knowledge of heart complications in diabetes.

A 2018 study conducted in Makkah, Saudi Arabia [14], found that even though the general knowledge of diabetic complications was high (80%), the specific knowledge about heart disease risk in diabetes was only 40.1%.

Acknowledging the knowledge deficit of the increased risk of developing CVDs in the diabetic population could assist in designing and implementing better educational programmes to improve long-term glycaemic control, as well as in adherence to medication and lifestyle changes among patients with diabetes. Consequently, this could help in decreasing the incidence of heart disease among patients with type 2 diabetes mellitus (DM). However, few studies have discussed the CVD risk knowledge in Saudi Arabia, especially among patients with type 2 DM. Therefore, the purpose of this study was to assess the level of CVD risk knowledge among adults with type 2 diabetes and identify factors that might be associated with the knowledge deficit.

## 2. Materials and Methods

### 2.1. Study Design and Population

A cross-sectional study was conducted from September 2019 to April 2020 in the primary care outpatient clinics, family medicine clinics, and VIP clinics at King Saud University Medical City (KSUMC), a tertiary hospital in Riyadh, the capital of Saudi Arabia. A convenience sampling method was used to recruit the study participants. The inclusion criteria were as follows: patients with type 2 DM, Saudis who were older than 18 years of age, and patients visiting the outpatient clinics in KSUMC. The exclusion criteria were as follows: patients with type 1 DM or gestational diabetes, patients less than 18 years of age, non-Saudis, severely ill patients, and patients with mental illness.

Written informed consent was obtained from all the participants. The study was approved by the local research ethics committee with the approval number E-19-4443.

### 2.2. Sample Size Estimation

The sample size was calculated using the single mean formula: (*n* = Zα2 S2/d2), where Zα = 1.96 (assuming 95% level of confidence), S = 2.83 (according to the results of a similar previous study [15]), and d = 0.3 (by calculating a precision of 10% of the standard deviation (SD) (2.83 × 0.10 = 0.3)).

Using the above formula, the calculated sample size by anticipating a 10% non-response rate was 377.

### 2.3. Data Collection

Data were collected using a paper-based questionnaire. The questionnaire included socio-demographic variables (age, gender, income, level of education, marital status, type of house, and place of residence). It also consisted of information on diabetes-related factors (duration of DM, family history of diabetes, and diabetes regimen). The next part of the questionnaire comprised an Arabic-translated version of the Heart Disease Fact Questionnaire (HDFQ) [16]. HDFQ is a validated questionnaire of 25 items for measuring heart disease risk knowledge, with possible answers of ‘true’, ‘false’, and ‘I do not know’. The scores were calculated by giving one point for each correct answer and zero points for incorrect answers or ‘I do not know’ responses. The total knowledge score was calculated by summing the points for the correct answers and then grading the score out of 25.

### 2.4. Statistical Analyses

Data were analysed using Statistical Package for the Social Science software (SPSS 24.0; SPSS Inc., Chicago, IL, USA). Descriptive statistics were used to describe the quantitative (mean [M] ± SD) and categorical variables (frequency and percentage). A *p*-value of <0.05 and a 95% confidence interval were used to report the statistical significance and precision of the results. The internal consistency coefficient of the Arabic version of the questionnaire was measured using the Kuder–Richardson 20 test.

## 3. Results

### 3.1. Participant Characteristics

Of the 453 distributed questionnaires, 383 (84.5%) participants with type 2 DM responded. Approximately 51.7% of the participants were female, 35.8% were in the age group of 60 to 70 years, approximately 77% were married, and 97.6% resided in urban areas. The socio-demographic characteristics of the study participants are presented in Table 1.

Most participants had type 2 diabetes for an average of 5 years or less (24.9%), only used oral hypoglycaemic agents to control their diabetes (65.3%), and had a family history of one or both parents with DM (67.9%) (Table 2).

### 3.2. Heart Disease Fact Questionnaire (HDFQ)

The Arabic version of the HDFQ used in this study showed good internal consistency (0.73) on the Kuder–Richardson 20 test. The mean knowledge of heart disease was generally high (19.04 ± 3.47), ranging from 6 to 25. Item analysis revealed knowledge deficits (items that less than 70% of the respondents had answered correctly) related to the knowledge of good and bad cholesterol, the effect of gender and family history on the risk of developing heart disease, and whether individuals are always aware that they have heart disease (Table 3).

### 3.3. Knowledge of Heart Disease Score among Different Groups

The HDFQ scores were not associated with gender, place of residence, educational level, or gross monthly income. The scores did not differ according to the duration of diabetes, diabetes regimen, or positive family history of diabetes.

A one-way analysis of variance test revealed a significant effect of age on the HDFQ score at the *p* < 0.05 level for the different age groups (F (2,38) = 5.65, *p* = 0.01). Post hoc comparisons using the Tukey Honest Significant Difference test indicated that the mean score for the participants who were 40 years of age or younger (M = 17.62, SD = 3.78) was significantly lower than those of participants aged 40 to 60 years (M = 19.37, SD = 3.52) and older than 60 years (M = 19.10, SD = 3.14). This indicated an overall increase in the HDFQ score with increase in age. The test was also significant for social status (F (2,38) = 4.92, *p* = 0.01), with a significant increase in the mean score of the married group (M = 19.35, SD = 3.44) when compared with those in the widowed or divorced group (M = 18.03, SD = 3.46). Furthermore, the effect of the type of house on HDFQ score was significant (F (1,38) = 2.77, *p* = 0.04). The participants living in traditional houses (M = 19.89, SD = 2.03) scored the highest followed by those living in villas (M = 19.28, SD = 3.52), then those living on floors (M = 17.90, SD = 3.21) and finally those living in apartments (M = 18.91, SD = 3.52) (Table 4).

## 4. Discussion

This study aimed to investigate the CVD risk knowledge in adults with type 2 DM visiting KSUMC outpatient clinics in Riyadh, Saudi Arabia. Knowledge of CVDs risk was high among the participants in this study (19.026 ± 3.47). However, in the study conducted in Turkey using the same questionnaire, the mean score was only (15.7 ± 4.4) [3]. A 2007 study conducted in Oman [17] found that only 20.4% of the population considered heart disease as a possible complication of diabetes. This was consistent with multiple studies conducted from 2009 to 2016 [7,12,13,18]. Furthermore, in a 2018 study conducted in Makkah, Saudi Arabia [14], the percentage of the participants having CVD risk knowledge was only 40.1%. Higher scores in the study sample can be attributed to the ‘multidisciplinary intensive education programme aimed at the glycaemic control and CVD risk factors of type 2 DM patients’ that is being conducted at KSUMC since April 2014 [19]; the programme provides personal education about diabetic complications and CVD risk reduction among many other services. The difference in results in the Makkah study might have been due to the use of a different survey tool and the large diversity in ethnic backgrounds in the western region.

The study results for the predictors of the HDFQ scores showed a significant association (*p* = 0.004) between age and higher knowledge scores. Participants above 40 years of age had better CVD risk knowledge, and hence, they scored higher. These results were consistent with those of a study conducted in Dhaka, Bangladesh [12]. Conversely, the Turkish study had proposed that younger participants would score better than older participants [3]. However, it should be considered that older patients would show a better attitude toward gaining information on heart disease. This attitude, which is mostly driven by fear of ill health with aging, might have contributed to better knowledge scores [20].

The results of social status were in line with those of previous studies [7,18], demonstrating that marital status predicted the knowledge score (*p* = 0.008). The study conducted in Ghana [7] revealed that married participants were three times more likely to have an increased understanding of diabetic complications. A systematic review conducted to evaluate the determinants affecting health care behaviour among diabetics concluded that guidance from the society and families on the patients’ meals, appointments, and medications had a positive effect on the patients which led to better management of diabetes [21].

To the best of our knowledge, no epidemiological studies have assessed the relationship between the type of house of the patient and their CVD risk knowledge score. In this study, housing type was a predictor of the knowledge score (*p* = 0.042). The participants living in traditional houses scored higher than those living in villas did. Although the findings showed a significant association between the type of housing and the CVD risk knowledge score, these results need further clarification in future studies due to the very small number of participants living in traditional houses (*n* = 9) in comparison with those living in villas (*n* = 262).

In contrast to our study results, many studies have found positive correlations between a higher level of education and the attainment of higher knowledge scores [3,7,17,18]. The insignificant results in our study (*p* = 0.259) showed that the level of education did not influence the degree of knowledge.

The HDFQ scores in our study were not correlated with the duration of the disease, which was contradictory to the results of other studies [7,22]. Our study results were more similar to those of the Turkish study [3] which hypothesised a possible behavioural change among patients living with diabetes for a longer time, such as the belief that they were capable of self-management of the disease without the need to receive training/checkup. Consequently, they might not obtain sufficient knowledge or retain the acquired information.

The results indicated no significant association between family history and the attainment of higher knowledge scores (*p* = 0.255). This was in contrast to the results of the study conducted in Oman [17] that showed a correlation between a higher level of knowledge and positive family history. Our results were more in line with those of the study in Dhaka [12]. The authors of that study suggested that although receiving information from a family member with a chronic disease might influence the patient’s attitude and daily practice, such informal sources are not always reliable. In support of this view, Pierce et al. [23], in their randomised controlled trial, found that family members of individuals with type 2 diabetes underestimated their risk of developing the disease.

Overall, the results of our study showed a high level of knowledge of CVD risk among all participants. However, despite the high level of knowledge, the prevalence of CVD remains high at 24% among the Saudi population [24]. This finding can be attributed to the suboptimal levels of adherence to medication among patients [25,26]. Assessing different dimensions such as patients’ beliefs and attitudes as well as applying different behavioural theories [27] might help elucidate the disparity between knowledge of CVD risk and medication adherence in the population.

Furthermore, the recent upsurge in information technology and social media usage has created a platform where falsehood spreads faster than truth [28], where there are many false sources of health information [29]. Considering this, ensuring that patients receive information from verified sources might be the key to combating misinformation and improving CVD risk knowledge levels. Therefore, assessing sources of information as well as adding open-ended questions to evaluate a patient’s current knowledge might contribute to this goal.

In contrast to previous studies, high levels of knowledge in our study population, despite low levels of medication adherence reported in the literature, imply that a shift in focus needs to be made from patient education towards exploring other aspects of behaviour, such as patients’ attitudes, beliefs, and risk perceptions, and identifying the best course of action to improve medication adherence and decrease the prevalence of cardiovascular events. 

The study has a few limitations. First, it included diabetic patients who visited KSUMC only. However, this is a tertiary referral hospital that treats people from different areas in the country. Another limitation is that the HDFQ used in this study consisted mainly of questions that assessed knowledge by presenting a ‘yes/no’ type of question. To display a superior level of knowledge, some participants tended to reply with ‘yes’ to every question posed. Thus, this might have introduced a degree of response bias. However, the participants were given constant encouragement by the interviewers to choose the option ‘I do not know’ whenever applicable. Additionally, although the questionnaire was self-administered, most participants (77.5%) chose to have the survey be read to them, which might have introduced a degree of interviewer bias.

## 5. Conclusions

The study results highlighted a high level of CVD risk knowledge in our study population, particularly among patients who were older, married, and lived in traditional houses. The presence of multidisciplinary intensive education programmes targeting type 2 DM patients, such as that present at KSUMC, might be responsible for the higher levels of CVD risk knowledge among our study population.

## Figures and Tables

**Table 1:** Socio-demographics characteristics of the study participants (*n* = 383).

Characteristics	*n* (%)
**Gender**	
Male	185 (48.3)
Female	198 (51.7)
**Age (years)**	
≤30	23 (6.0)
30–39	25 (6.5)
40–49	54 (14.1)
50–59	120 (31.3)
60–70	137 (35.8)
>70	24 (6.3)
**Place of residency**	
Urban	369 (97.6)
Rural	9 (2.4)
Missing	5 (1.32)
**Educational level**	
Not educated	73 (19.1)
Can read and write	18 (4.7)
Primary school	55 (14.4)
Intermediate school	42 (11.0)
High school	74 (19.3)
Bachelor’s degree	102 (26.6)
Post-graduate	19 (5.0)
**Social statuses**	
Married	295 (77)
Divorced	20 (5.2)
Unmarried	21 (5.5)
Widow	42(11.0)
Missing	5 (1.3)
**Housing**	
Villa	262 (68.4)
Floor	59 (15.4)
Apartment	53 (13.8)
Traditional house	9 (2.3)
**Gross monthly income (in Saudi Riyals)**	
<5000	129 (33.7)
5000–8000	66 (17.2)
8001–10,000	49 (12.8)
10,001–15,000	60 (15.7)
15,001–20,000	51 (13.3)
>20,000	18 (4.7)
Missing	10 (2.6)

**Table 2:** Diabetes-related characteristics of the study participants (*n* = 383).

Diabetic Characteristics	*n* (%)
**Duration of diabetes (years)**	
≤5	114 (29.8)
5–10	94 (24.5)
11–15	59 (15.4)
16–20	51 (13.4)
>20	63 (16.5)
Missing	2 (0.52)
**Diabetes regimen**	
Diet only	14 (3.7)
Oral agents	250 (65.3)
Insulin injections	24 (6.3)
Oral agents and insulin injections	95 (24.8)
**Family history of diabetes**	
Yes	260 (67.9)
No	123 (32.1)

**Table 3:** Heart Disease Fact Questionnaire items with less than 70% correct response.

Item	Frequency Corrects	Percent Correct
A person always knows when they have heart disease.	142	37%
If you have a family history of heart disease, you are at risk for developing heart disease.	199	51.9%
If your ‘good’ cholesterol (HDL) is high you are at risk for heart disease.	151	39.4%
People with diabetes rarely have high cholesterol.	230	60%
People with diabetes tend to have low HDL (good) cholesterol.	154	40%
Men with diabetes have a higher risk of heart disease than women with diabetes.	153	39.9%

**Table 4:** Sample population characteristics with analysis of variance tests for knowledge scores.

Characteristics	CVD Knowledge Total (Min = 0; Max = 25)			
	Mean	SD	F	*p*-Value
Gender *			0.07	0.79
Male	19.08	3.44		
Female	18.98	3.52		
**Age (years)**			5.65	**0.01**
≤40	17.62	3.78		
40–60	19.37	3.52		
>60	19.10	3.14		
Place of residency *			0.02	0.89
Urban	19.05	3.48		
Rural	18.89	3.22		
**Educational level**			1.35	0.26
Not educated	18.59	3.30		
Can read and writes, primary	18.58	3.59		
Intermediate, high school	19.40	3.22		
Bachelor’s degree, postgraduate	19.21	3.71		
**Social status**			4.92	**0.01**
Married	19.35	3.44		
Divorced, widow	18.03	3.46		
Unmarried	17.95	3.43		
**Housing**			2.77	**0.04**
Villa	19.28	3.52		
Floor	17.90	3.21		
Apartment	18.91	3.52		
Traditional house	19.89	2.03		
**Gross monthly income (in Saudi Riyals)**			1.48	0.21
<5000	18.84	3.40		
5000–8000	18.38	2.79		
8001–10,000	19.04	4.02		
10,001–15,000	19.72	3.59		
>1500	19.41	3.54		
**Duration of diabetes (years)**			0.41	0.80
≤5	19.00	3.48		
5–10	19.42	3.35		
11–15	19.09	3.73		
16–20	18.98	3.87		
>20	18.73	2.78		
**Diabetes regimen**			0.27	0.85
Diet only	19.43	4.48		
Oral agents	19.11	3.50		
Insulin injections	18.79	2.60		
Oral agents and insulin injections	18.81	3.46		
Family history of diabetes *			0.68	0.41
Yes	19.13	3.59		
No	18.81	3.20		

* Independent *t*-test.

## Data Availability

All data generated or analysed during this study are included in this published article.
